# From retention to turnover: the paradoxical role of career optimism and the moderating effect of psychological safety

**DOI:** 10.3389/fpsyg.2025.1706367

**Published:** 2026-01-14

**Authors:** Zhenxing Gong, Yue Lu, Miaomiao Li

**Affiliations:** 1 School of Business, Liaocheng University, Liaocheng, China; 2 Business School, Beijing Information Science and Technology University, Beijing, China

**Keywords:** career optimism, job-seeking behavior, organizational commitment, psychological safety, subjective career success

## Abstract

This study aims to explore the dual effects of career optimism on job-seeking behavior and organizational commitment, clarify the underlying mechanisms, and provide organizational management strategies to maximize benefits and mitigate drawbacks. However, the relationship between career optimism, organizational commitment and job-seeking behavior lacks consistency. Using cluster sampling, 370 employees were surveyed at two time points. Bootstrap and moderated mediation analyses were applied to test hypotheses, based on Social Cognitive Career Theory. An analysis of data from 370 employees over two periods found that: (1) Career optimism significantly and positively affected job-seeking behavior and organizational commitment; (2) Subjective career success mediated the relationship between career optimism and job-seeking behavior/organizational commitment; (3) Psychological safety moderated the relationship between subjective career success and job-seeking behavior, that is, the lower the level of psychological safety, the higher are the positive relationship of subjective career success and job-seeking behavior. Theoretically, it reveals the “dual-path” effect of career optimism, and supplements the explanatory gap of Social Cognitive Career Theory in situational moderating mechanisms. Practically, it offers strategies for organizations to balance optimism’s benefits and risks, aiding talent retention.

## 1 Introduction

The global professional landscape is undergoing profound transformation, driven by accelerated digitalization, deep industrial restructuring, and increasingly flexible employment relationships. These forces collectively create unprecedented complexity and uncertainty within individual career development, making employees’ career attitudes and behavioral choices a central concern in organizational management research.

Amidst profound shifts in the contemporary workplace, career optimism—a core dimension of positive psychological capital defined as an individual’s positive expectations and outlook toward achieving optimal future career outcomes (Rottinghaus et al., 2005)—has gained prominence as a pivotal factor in fostering proactive career behaviors and sustaining organizational commitment. As a domain-specific manifestation of dispositional optimism, career optimism aligns with Carver and colleagues’ conceptualization of optimism as a generalized expectation of positive outcomes (Carver et al., 2010). It encompasses both persistent goal-directed effort and positive outcome expectations, which are instrumental in navigating career-related challenges. However, the effects of career optimism are not uniformly adaptive. Recent research by Shamir-Balderman et al. (2024) highlights this paradoxical effect. In contexts marked by high uncertainty—a defining feature of modern careers—career optimism may heighten anxiety and trigger preemptive job mobility among younger workers. Building on these insights, this study investigates how this core psychological trait interacts with organizational conditions to shape consequential career behaviors.

Viewed through the broader theoretical lens of Psychological Capital (PsyCap), as a higher-order construct comprising self-efficacy, optimism, hope, and resilience, career optimism emphasizes that positive psychological states serve as key resources driving individual attitudes and behaviors. Career optimism aligns with humanistic and boundaryless career perspectives (Duffy, 2010) and resonates with the principles of positive psychology as well as ethos of contemporary stable societal contexts. As a career-related attitude and belief, its influence on career behaviors, cognitions, and competencies has attracted widespread theoretical interest (Eva et al., 2020).

However, despite its theoretical appeal, recent research has revealed that the effects of career optimism are not entirely positive and may even entail a notable “double-edged sword” effect. For instance, Eva et al. (2020) observed that excessive career optimism may lead to turnover intentions, as individuals persistently strive after the illusion of a “better next job.” Even in the absence of objective constraints (e.g., skill deficits or career plateau), highly optimistic employees may perceive non-existent constraints which can dilute focus and impair in-role performance. Further complicating this picture, Duffy and Raque-Bogdan (2010) found that although career-optimistic individuals perceive more career options, such an abundance can paradoxically make career decision-making more challenging. These contradictory findings present a managerial dilemma: while fostering optimism may strengthen organizational identification, it also risks stimulating frequent job-seeking behavior, thereby undermining team stability and long-term organizational health. The present study empirically confirms that career optimism significantly predicts both job-seeking behavior and organizational commitment, supporting the coexistence of these dual effects.

Current literature explains these contradictory effects primarily through three lenses: the “too much of a good thing” effect (Dillard et al., 2009), boundary conditions such as neuroticism and tolerance for uncertainty (Kim et al., 2016), and the “double-edged sword” effect (Eva et al., 2020). However, much of the literature remains confined to singular theoretical perspectives such as Social Cognitive Career Theory (SCCT) or positive psychology, and has yet to systematically uncover the cognitive appraisal mechanisms through which career optimism simultaneously shapes divergent behavioral outcomes such as job search and organizational commitment.

This study identifies subjective career success—defined as an individual’s internal evaluation of career accomplishments against personal standards—as a critical mediating mechanism that translates optimistic beliefs into action. Specifically, career optimism enhances perceived subjective career success, which in turn fosters both external opportunity exploration (job-seeking behavior) and internal loyalty reinforcement (organizational commitment).

More importantly, we clarify the moderating role of psychological safety—employees’ sense of security in taking interpersonal risks (Edmondson, 1999) which refers to a shared belief that one can express oneself freely without fear of negative consequences to self-image, status, or career. In environments characterized by low psychological safety, the positive relationship between subjective career success and job-seeking behavior is significantly strengthened, generating a paradoxical “escape advantage” rather than fostering “stay motivation.” This finding challenges the conventionally positive view of psychological safety and reveals a protective motivation mechanism that is activated precisely in its absence—aligning with Kahn’s (1990) early work on psychological safety as a prerequisite for engagement. Psychological safety refers to a shared belief that one can express oneself freely without fear of negative consequences to self-image, status, or career (Edmondson, 1999). It fosters a climate of openness, encouraging mindfulness practices, sharing of innovative ideas, and commitment to organizational goals (Ming et al., 2015). Based on Social Exchange Theory, reciprocal processes triggered by benevolent acts cultivate trust and psychological security, which in turn influence critical outcomes such as job satisfaction (Mitterer and Mitterer, 2023). Research indicates that psychological safety acts as an antecedent to trust, which mediates the relationship between psychological safety and job satisfaction, offering critical insights into the micro-mechanisms through which psychological safety influences subsequent attitudes and behaviors (Ming et al., 2015; Mitterer and Mitterer, 2023).

It is also important to recognize that organizational-level human resource management systems significantly shape employees’ cognitive and behavioral patterns (Simó Guzmán et al., 2010). Studies indicate that high-commitment work systems (HCWS)—characterized by internal promotion, extensive training, and participatory decision-making—enhance employees’ skills, motivation, and organizational identification. These systems thereby strengthen self-efficacy and perceptions of career prospects, ultimately promoting proactive behaviors (Shi and Cao, 2022; Tang et al., 2019). Similarly, research on psychological capital (PsyCap) indicates that positive psychological states (e.g., hope, resilience) significantly improve job satisfaction and organizational commitment, providing additional theoretical support for understanding the pathways through which positive cognitive resources (e.g., career optimism) influence commitment. Both HCWS and PsyCap help cultivate long-term psychological bonds between employees and organizations, thereby serving as key contextual factors that reinforce positive work-related attitudes (Shi and Cao, 2022). Furthermore, subjective career success is not only a matter of personal achievement perception but also closely linked to organizational commitment. Simó Guzmán et al. (2010) found that subjective career success positively affects affective commitment and negatively influences continuance commitment, reflecting the broader shift in psychological contracts from relational to transactional terms. This trend underscores the growing importance employees place on intrinsic fulfillment and self-directed career development (Shi and Cao, 2022). Together, these insights provide evidence for understanding the mechanism through which career optimism influences commitment via subjective career success.

Therefore, based on Social Cognitive Career Theory (Lent et al., 1994) and integrating perspectives from Self-Determination Theory and Social Exchange Theory, this study aims to address three core questions:

Is employee career optimism purely beneficial, or does it possess dual (promotive/inhibitory) attributes for organizations?

If a dual effect exists, what is the underlying mechanism? Does subjective career success play a key mediating role?

How do contextual factors, such as psychological safety (and the trust it may engender), moderate these mechanisms? What management strategies can organizations employ to maximize benefits and mitigate risks?

To address these complex questions, this study develops an integrated theoretical framework grounded in Social Cognitive Career Theory (SCCT), Social Exchange Theory, and Self-Determination Theory (SDT). SCCT provides the foundational “cognition-behavior” pathway, explaining how career optimism influences behavior through subjective evaluations (i.e., subjective career success). However, SCCT alone cannot adequately explain why the same perception of success may lead to divergent behavioral outcomes: organizational retention versus turnover intention. Social exchange theory offers a critical supplement here. It suggests that when employees attribute their subjective career success to organizational support, they tend to strengthen their organizational commitment based on the norm of reciprocity. Conversely, in the absence of such attribution, they may seek equivalent exchange relationships elsewhere, thereby increasing job-seeking behavior.

Meanwhile, Self-Determination Theory provides further nuance by positing that the translation of positive cognitions into autonomous motivation depends on whether the environment satisfies individuals’ basic psychological needs for autonomy, competence, and relatedness. Psychological safety, as a key contextual factor that fulfills these innate needs, is thus positioned as a moderating variable that determines whether the experience of subjective career success is channeled into internal loyalty and retention or external opportunity exploration.

**FIGURE 1:**
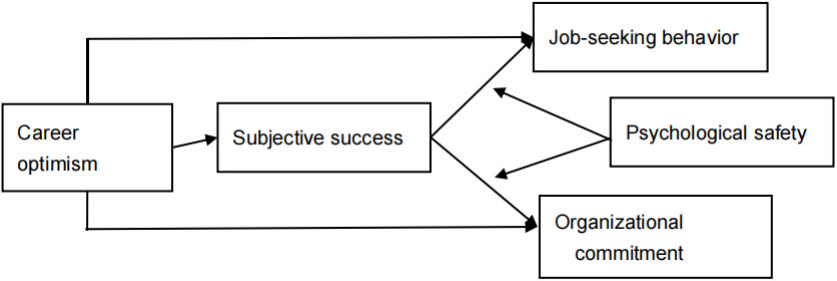
Theoretical model diagram.

By integrating these theoretical perspectives, this study offers a nuanced explanation for the paradoxical effects of career optimism—one that cannot be attained through any single theoretical lens. In the following sections, we systematically elaborate on the conceptual model and empirical design based on a thorough literature review and hypothesis development. The study’s theoretical model is depicted in Figure 1.

## 2 Literature review and hypothesis development

### 2.1 Career optimism, job-seeking behavior, and organizational commitment

Job-seeking behavior is typically defined as proactive efforts by individuals to identify, evaluate, and secure new job opportunities (Boswell et al., 2003). Individuals with high career optimism focus on their interests and future career development. They prepare for their envisioned future selves (e.g., through observation and learning) and feel they are progressing toward career success (Gunkel et al., 2010; Haratsis et al., 2015). Social Cognitive Career Theory (SCCT) posits that outcome expectations, such as career optimism, directly influence goal-directed behavior (Lent et al., 1994). Employees with high career optimism possess strong confidence in their future career prospects. This belief drives them to engage in exploratory actions to identify potential opportunities. Career optimism reinforces the belief that job-seeking behavior will lead to desirable outcomes (e.g., promotion, ability-job fit, value realization) (Rottinghaus et al., 2005), thereby increasing their effort investment in job search (Kanfer et al., 2001). Optimists are more likely to perceive job search obstacles as challenges to overcome rather than threats, enhancing their persistence in the face of setbacks (Lent, 2013). Empirical studies confirm that dispositional optimism is positively correlated with job search intensity and job search self-efficacy (Zikic and Klehe, 2006). Modern careers emphasize the need for employees to proactively manage their career trajectories (Hall, 2002). Employees with high career optimism are thus more likely to engage in continuous job search activities (e.g., updating resumes, attending interviews) to maintain “marketability” and achieve long-term career goals (Tolentino et al., 2013). Career optimism embodies positive expectations. Individuals holding favorable outcome expectations typically believe they can significantly influence employment success. They tend to address stressful problems through active individual action and are more likely to invest time and energy in job search (Kanfer et al., 2001). They also tend to develop systematic career plans for their future and proactively explore new job opportunities to expand career possibilities (El-Anzi, 2005). Collectively, these characteristics indicate that the positive expectations inherent in career optimism not only shape individuals’ perceptions of employment outcomes but also drive proactive behavioral tendencies in career development, manifested as agency in problem-solving and foresight in career planning.

*H1*: Career optimism has a significant positive effect on job-seeking behavior.

Organizational commitment refers to an individual’s identification with, and trust in, the goals and values of their employing organization, along with the resulting positive affective attachment. SCCT suggests that outcome expectations influence performance goal levels, with stronger expectations leading to higher aspirations (Karrasch, 2017), thereby promoting more stable and persistent task behavior. Career optimism, as a robust predictor of persistent goal pursuit, includes expectations of favorable outcomes even in the face of potential adversity.

When employees possess high career optimism and believe they can achieve their career goals within the organization, they perceive the organization as a vehicle for self-actualization, thereby enhancing their affective commitment (Meyer et al., 2004). Furthermore, as a psychological resource, career optimism helps employees positively interpret their organizational environment (Hobfoll, 1989), making them more likely to perceive organizational support and development opportunities. This fosters a sense of obligation to reciprocate (normative commitment) and sensitivity to the costs of leaving (continuance commitment) (Meyer and Maltin, 2010). Additionally, career optimists cope effectively with stress (Chemers et al., 2000; Gillham, 2000) and proactively build linkages with the organization (e.g., skill matching). This strengthens commitment through a “fit-link-sacrifice” mechanism (Ng et al., 2005). Empirical research also indicates a significant positive correlation between career optimism and organizational commitment (Magalhães, 2015)

*H2*: Career optimism has a significant positive effect on organizational commitment.

### 2.2 Career optimism and subjective career success

Subjective career success is defined as an individual’s subjective perception of their human capital reserves, self-worth, and career satisfaction (Ng et al., 2005), moving beyond traditional objective measures like salary and promotion. Employees with high career optimism hold positive expectations about their future career development (Rottinghaus et al., 2005). This optimism fosters subjective career success through multiple pathways: Optimists set higher-order career goals aligned with their values (e.g., skill mastery, increased influence), not just short-term performance targets. They attribute career progress to internal, stable factors (ability, effort) rather than external circumstances or luck (Gillham, 2000), enhancing feelings of control and satisfaction (Bretz Boudreau and Judge, 1994). Aligned with SCCT (Lent, 2013), they proactively seek developmental challenges, pursue high-value tasks, and secure training resources (Ng et al., 2005), directly boosting perceptions of competency growth (Guan et al., 2015). They actively build supportive networks for information and referrals (Wolff and Moser, 2009), enhancing perceptions of organizational influence and social recognition (Heslin, 2005).

Career optimism cultivates positive self-evaluations, including perceived career adaptability (Duffy and Raque-Bogdan, 2010), career decision clarity (Gunkel et al., 2010), and self-efficacy (Kim et al., 2016). Optimists exhibit greater resilience, self-control, and persistence (Eva et al., 2020) and are more prepared and willing to adapt to changing environments (Bravo et al., 2017), thereby enhancing subjective career success.

*H3*: Career optimism has a significant positive effect on subjective career success.

### 2.3 Subjective career success, job-seeking behavior, and organizational commitment

In an unpredictable career landscape, individuals experiencing subjective career success focus on enhancing competencies to navigate external challenges. They value mobility across roles, departments, and organizations to achieve goals. Success enhances confidence in market value (Ng et al., 2005), prompting proactive job-seeking behavior to evaluate external opportunities and maximize career resource returns (Seibert et al., 2016). Boundaryless career mindsets shift motivation (Zacher, 2014). Subjectively successful individuals pursue challenging goals (e.g., cross-organizational moves, role transitions) (Abele and Spurk, 2009), where job search becomes a necessary tool (Zikic and Klehe, 2006). Positive self-evaluations strengthen beliefs in controlling the career environment (Bretz Boudreau and Judge, 1994), thereby driving proactive environmental scanning to maintain advantage (Boswell et al., 2012)

*H4*: Subjective career success has a significant positive effect on job-seeking behavior.

Achieving subjective career success within an organization signifies internal career capital and competitive advantage. Leaving risks losing this platform for growth and goal achievement, imposing high costs. To protect and build upon this success, individuals value their established position, increasing organizational commitment. Success fosters intrinsic task engagement and identification (Guan et al., 2015). Reciprocity norms also lead employees to repay the organization for enabling their value growth and positive self-affirmation, strengthening affective bonds.

*H5*: Subjective career success has a significant positive effect on organizational commitment.

SCCT emphasizes the interaction between environment, cognition, and behavior. Career optimism, as a key cognitive belief, enhances subjective career success by fostering perceptions of internal recognition. Attributing this success to the organization strengthens commitment. Conversely, optimism also drives external career planning and mobility to achieve broader aspirations.

*H6*: Subjective career success mediates the relationship between career optimism and job-seeking behavior.

*H7*: Subjective career success mediates the relationship between career optimism and organizational commitment.

### 2.4 Psychological safety, job-seeking behavior, and organizational commitment

Psychological safety refers to an individual’s perception that they can freely express themselves without fear of negative consequences for their image, status, or career (e.g., job uncertainty and interpersonal risk) (Edmondson, 1999). Social cognitive theory suggests that attitudes and behaviors are influenced by personal needs, goals, and environmental cues, providing a theoretical basis for understanding the role of psychological safety.

When employees perceive a high level of psychological safety, they are more likely to voice their opinions, engage in social exchange with the organization, and strengthen their organizational identification, thereby enhancing organizational commitment and reducing turnover intention (Singh et al., 2013). Empirical studies confirm that employees with high psychological safety exhibit stronger retention intentions and lower turnover intentions. Theoretically, prior research grounded in social exchange theory posits that psychological safety encourages employees to reciprocate with attitudes and behaviors desired by the organization (Frazier et al., 2017). Consistently, psychological safety has been found to positively correlate with favorable work attitudes such as organizational commitment (Newman et al., 2017).

High psychological safety can also mitigate the negative correlation between achievement orientation and voice role encapsulation, empowering individuals to speak up, enhancing their sense of team integration, and strengthening organizational commitment (Tangirala et al., 2013). Moreover, psychological safety helps employees maintain positive affect, perceive external situations optimistically, and reduce the psychological pressure to avoid negative outcomes, thereby reinforcing positive behavioral tendencies (Frazier et al., 2017), which in turn strengthens organizational commitment.

Conversely, when psychological safety is lacking, employees tend to avoid risks, reduce positive interactions with the organization, and may increase job-seeking behavior. Furthermore, a context of high psychological safety encourages learning new skills and proposing novel ideas, whereas a low-safety context inhibits the application of skills (Frazier et al., 2017). Such differences further influence employees’ attachment to the organization, namely their level of organizational commitment versus their tendency toward job-seeking behavior.

More importantly, psychological safety is likely to play a key moderating role in the process through which subjective career success translates into job-seeking behavior. Specifically, we expect psychological safety to weaken the positive effect of subjective career success on job-seeking behavior:

According to job embeddedness theory, an environment high in psychological safety embeds employees more deeply within the organization by strengthening their links, fit, and sacrifices. Consequently, even when employees perceive high subjective career success, their deep embeddedness reduces the motivation to convert such perceptions into actual job-seeking actions.

Conservation of Resources theory offers a complementary perspective: in a high-safety environment, employees do not need to expend extra resources to manage interpersonal risks or self-censorship. Active job-seeking is itself perceived as a high-cost resource expenditure (Hobfoll, 1989). Thus, employees with high subjective career success are more inclined to invest their efforts in sustained development within the current organization rather than seeking external opportunities.

In contrast, in environments with low psychological safety, the embedding effect is weak, and employees continuously expend psychological resources to cope with interpersonal risks. In this case, the perceived market competitiveness derived from subjective career success is more directly translated into a protective motivation to “escape” the risky environment and seek a safer platform through job-seeking behavior.

Based on the above reasoning, we propose:

*H8*: Psychological safety moderates the relationship between subjective career success and job-seeking behavior. This relationship is weaker for employees with high psychological safety and stronger for those with low psychological safety.

*H9*: Psychological safety moderates the relationship between subjective career success and organizational commitment. This relationship is stronger for employees with high psychological safety and weaker for those with low psychological safety.

The strength of the indirect effects of career optimism (via subjective career success) on job-seeking behavior and organizational commitment depends on the employee’s level of psychological safety.

*H10*: Psychological safety moderates the indirect effect of career optimism on job-seeking behavior through subjective career success. This indirect effect is stronger when psychological safety is low and weaker when it is high.

*H11*: Psychological safety moderates the indirect effect of career optimism on organizational commitment through subjective career success. This indirect effect is stronger when psychological safety is high and weaker when it is low.

## 3 Research methods

### 3.1 Research procedure and sample

This study was approved by the Special Committee of Scientific Research Ethic of Liaocheng University (Approval No. HE2025051901).

This study employed cluster sampling to select 535 employees as the initial sample. To control for common method bias, questionnaires were administered at two time points, 3 months apart, following established practices. At Time 1, employees completed questionnaires measuring demographic variables, career optimism, and subjective career success. Three months later (Time 2), employees completed questionnaires measuring demographic variables, psychological safety, job-seeking behavior, and organizational commitment. A total of 370 valid matched questionnaires were obtained, yielding an effective response rate of 69.15%. The average age of the sample was 30.78 ± 9.54 years. Other demographic information is detailed in Table 1.

**TABLE 1:** Demographic characteristics of the participants.

Variable	Category	Frequency	Percentage (%)
Gender	Male	215	58.11
	Female	155	41.89
Female	155	41.89	
Education	Senior high school or below	92	24.86
	Junior college	45	12.16
	Bachelor’s degree	190	51.35
	Postgraduate or above	43	11.62
Work experience	5 years or less	218	58.92
	6–10 years	41	11.08
	11–15 years	44	11.89
	16–20 years	27	7.30

In this study, a “cluster” refers to an intact department comprising multiple collaborating enterprises. The inclusion criterion required departments to have at least 15 members, ensuring sufficient internal variability and enabling meaningful assessment of team-level characteristics such as psychological safety. Through the research team’s university-based collaborative network and corporate contacts, human resource departments of several organizations were approached. After obtaining organizational-level consent, we requested a list of operational departments with relatively stable membership. From this list, eight departments were randomly selected as survey clusters. All members of the selected departments were invited to participate in the study.

The final sample consisted of eight distinct organizations, primarily from the information technology services sector (5 organizations, 62.5%) and manufacturing (3 organizations, 37.5%). This distribution reflects the contemporary talent dynamics in knowledge-intensive and technology-driven industries.

We assessed potential cluster effects using intraclass correlation coefficients (ICC 1). The results showed ICC (1) values of 0.07 for psychological safety, 0.05 for organizational commitment, and 0.04 for job-seeking behavior. All values fell below the conventional threshold of 0.12, indicating minimal between-group variance in the dependent variables and suggesting that the nested data structure did not significantly influence the results. Therefore, individual-level data analysis was deemed appropriate.

The study may be subject to self-selection bias. Organizations willing to participate in the research might place greater emphasis on employee experience and management practices, potentially resulting in an organizational climate that is more favorable than the industry average. Moreover, employees who completed both waves of the survey (valid response rate: 69.15%) may have been more conscientious or more engaged with career-related issues than those who dropped out. These factors could lead to slightly higher scores on positive psychological traits, such as career optimism, potentially overestimating the positive relationships among variables and limiting the generalizability of the findings to all types of organizations.

### 3.2 Measurement instruments

All scales used a 5-point Likert scale (1 = Strongly Disagree, 5 = Strongly Agree) unless otherwise specified.

Career optimism: Measured using the 11-item scale by Rottinghaus et al. (2005) (e.g., “I aspire to achieve my career ideals”). Responses ranged from 1 (Never) to 5 (Always). Cronbach’s α = 0.78.

Subjective career success: Measured using the 24-item scale by Shockley (e.g., “I find my work meaningful”). Cronbach’s α = 0.72 (Shockley et al., 2016).

Psychological safety: Measured using the 7-item scale by Edmondson (1999) (e.g., “It is safe to take risks and try new ideas in my team”). Cronbach’s α = 0.82.

Job-seeking behavior: Measured using a modified version of Blau’s (1994) 12-item scale, comprising two dimensions: Job Search Preparation (6 items) and Active Job Search (6 items). Given that the scale was developed nearly three decades ago, and the job-seeking environment and methods have undergone fundamental changes—particularly with the widespread adoption of the internet—we contextually adapted some of the items to ensure content validity. The revision followed the principle of “preserving original meaning while updating behavioral carriers.” For example, the original item “looked for job openings in newspapers” was modified to “searched for job vacancies online,” and “contacted the state employment service office” was updated to “contacted professional recruitment agencies or headhunters.”

The adaptation process was conducted independently by two researchers in organizational behavior, and a human resource management practitioner was invited to evaluate the revised items to ensure their representativeness and comprehensibility in the contemporary job-seeking context. Although a full confirmatory factor analysis (CFA) could not be performed due to sample size limitations, the revised scale demonstrated good internal consistency reliability (Cronbach’s α = 0.76) in this study. Moreover, the correlation patterns with theoretically related variables—such as career optimism and subjective career success—were consistent with expectations, providing preliminary support for the criterion-related validity of the adapted scale.

Organizational commitment: Measured using a 9-item short form of the scale by Mowday et al. (2013) (e.g., “I am proud to tell others that I am part of this organization”). Cronbach’s α = 0.97 (Mowday et al., 2013).

Control variables: Gender, age, education level, organizational tenure, and Openness to Experience (measured with 2 items from the Big Five personality inventory, Cronbach’s α = 0.85) were included as controls related to turnover. This study controlled for gender, age, education level, organizational tenure, and openness to experience. The selection of these variables was based on established theories of turnover and career development, as they have been consistently demonstrated to relate to employees’ turnover propensity and career attitudes.

## 4 Results

### 4.1 Discriminant validity test

To examine the discriminant validity among the key variables, confirmatory factor analysis (CFA) was conducted using Mplus 7.0. The six-factor model demonstrated good fit (χ^2^ = 99.3, *p* < 0.01; CFI = 0.97; TLI = 0.95; RMSEA = 0.07) and differed significantly from other models, indicating good discriminant validity among the main variables.

Following the recommendation of Podsakoff et al. (2003), the unmeasured latent method factor approach was employed to test for common method bias. The results showed that adding a method factor to the five-factor model (resulting in a six-factor model; χ^2^ = 95.00, *p* < 0.01; CFI = 0.97; TLI = 0.95; RMSEA = 0.07) did not yield significant improvement in model fit indices. Furthermore, CFA results indicated that the single-factor model had poor fit (χ^2^ = 191.00, *p* < 0.01; CFI = 0.92; TLI = 0.90; RMSEA = 0.10). Therefore, this study did not exhibit serious common method variance issues.

### 4.2 Descriptive statistics and correlation analysis

To mitigate the influence of extraneous variables on the research results, the categorical variables among the control variables (gender, education level, and organizational tenure) were dummy-coded. Pearson correlation analysis was then conducted using these three control variables along with age and Openness to Experience as controls. The results, presented in Table 2, show that:

**TABLE 2:** Means, standard deviations, and correlation coefficients of variables.

	*M* (Mean)	*SD* (Standard deviation)	Career optimism	Subjective career success	Psychological safety	Organizational commitment	Job-seeking behavior
1. Career optimism	3.64	1.05	–				
2. Subjective career success	3.65	0.88	0.65^**^	–			
3. Psychological safety	3.28	0.91	0.28^*^	0.37^*^	–		
4. Organizational commitment	3.73	1.01	0.62^**^	0.64^**^	0.44^**^	–	
5. Job-seeking behavior	3.84	0.97	0.63^**^	0.72^**^	0.35^*^	0.61^**^	–

*p < 0.05,

**p < 0.01.

Career optimism was significantly positively correlated with subjective career success, psychological safety, organizational commitment, and job-seeking behavior (*p* < 0.01).

Subjective career success was significantly positively correlated with organizational commitment and job-seeking behavior (*p* < 0.01).

Psychological safety was significantly positively correlated with organizational commitment and job-seeking behavior (*p* < 0.01).

The detailed effects of the control variables across all models are presented in Table 3.

**TABLE 3:** Effects of control variables in main regression models.

Variable	Model 1 (SCS)	Model 2 (JSB)	Model 3 (JSB)	Model 5 (OC)	Model 6 (OC)
Gender	0.08	0.06	0.04	0.07	0.05
Age	0.12*	0.09	0.07	0.14*	0.11*
Education	0.05	0.03	0.02	0.06	0.04
Tenure	−0.10	−0.08	−0.06	−0.09	−0.07
Openness	0.15*	0.11	0.08	0.13*	0.10

SCS, Subjective Career Success, JSB, Job-Seeking Behavior, OC, Organizational Commitment. Gender: 0 = female, 1 = male; Education: 1 = high school or below, 2 = junior college, 3 = bachelor’s degree, 4 = postgraduate or above; Tenure in years.

*p < 0.05.

### 4.3 Hypothesis testing

**FIGURE 2:**
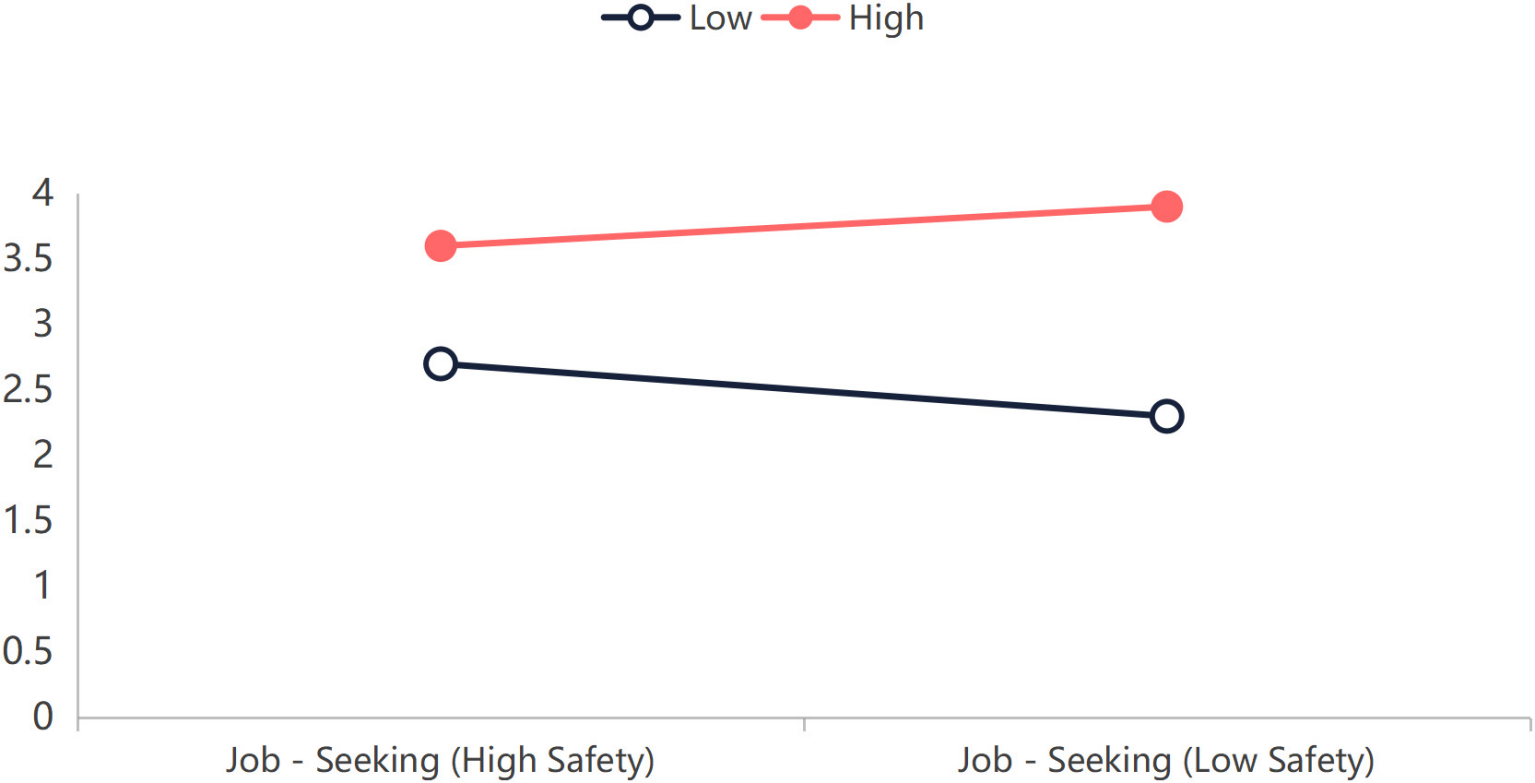
Moderating effect of psychological safety on the relationship between subjective career success and job—seeking behavior.

Mediation effects were tested using the Bootstrap method with 5,000 resamples. The findings are as follows:

(1)    Regression results in Table 4 show: In Model 1, career optimism had a significant positive effect on subjective career success (β = 0.37, *p* < 0.01). In Model 2, career optimism had a significant positive effect on job-seeking behavior (β = 0.45, *p* < 0.01). These results support Hypotheses 1 and 3. When both career optimism and subjective career success were included in the regression equation to predict job-seeking behavior (Model 3), subjective career success had a significant positive effect on job-seeking behavior (β = 0.58, *p* < 0.01), while the effect of career optimism on job-seeking behavior remained significant but decreased in magnitude (β = 0.23, *p* < 0.01). The mediation test results in Table 5: The indirect effect of career optimism on job-seeking behavior via subjective career success was 0.22, with a 95% confidence interval of [0.15, 0.29]. Since the interval does not include zero, the mediating role of subjective career success is confirmed. The mediation effect ratio was ab/c = 0.22/0.45 = 49%. These results support Hypotheses 4 and 6. Furthermore, in Model 4, the interaction effect of subjective career success and psychological safety on job-seeking behavior was significant (β = –0.10, *p* < 0.01), indicating that psychological safety moderates the relationship between career optimism and job-seeking behavior, supporting Hypothesis 8. Analysis of the control variables revealed that in regression models containing the main predictors, organizational tenure significantly and positively predicted organizational commitment (β = 0.09, *p*< 0.05). This finding aligns with expectations derived from the Investment Model, which posits that as employees’ tenure increases, their accumulated investments and perceived costs of leaving the organization rise, thereby enhancing their commitment. Simultaneously, openness to experience demonstrated a marginally significant positive relationship with job-seeking behavior (β = 0.08, *p* < 0.10), consistent with this personality trait’s association with exploration and novelty-seeking. The effects of other control variables—gender, age, and education level—were non-significant. These results substantiate the importance of controlling for these variables in the analysis.
(2)    Career optimism had a significant positive effect on organizational commitment (Model 5, β = 0.48, *p* < 0.01), supporting Hypothesis 2. After including both career optimism and subjective career success in the regression equation to predict organizational commitment (Model 6), subjective career success had a significant positive effect on organizational commitment (β = 0.42, *p* < 0.01), while the effect of career optimism on organizational commitment decreased but remained significant (Model 6, β = 0.33, *p* < 0.01). The indirect effect of career optimism on job-seeking behavior via subjective career success was 0.15, with a 95% confidence interval of [0.15, 0.23]. Since the interval does not include zero, the mediating role of subjective career success is confirmed. The mediation effect ratio was ab/c = 0.15/0.48 = 31%. These results support Hypotheses 5 and 7. Furthermore, the interaction effect of subjective career success and psychological safety on organizational commitment was not significant (Model 7, β = 0.03, ns), indicating that psychological safety does not moderate the relationship between career optimism and subjective career success. Hypotheses 9 and 11 were not supported.
(3)    To further analyze how psychological safety moderates the relationship between subjective career success and job-search behavior, a simple slope analysis was conducted (Dearing and Hamilton, 2006) As shown in Figure 2, for the group with low psychological safety (1 standard deviation below the mean), the effect of subjective career success on job-search behavior was relatively obvious (β = 0.64, *p* < 0.01), whereas for the group with high psychological safety (1 standard deviation above the mean), this relationship was significantly weaker (β = 0.46, *p* < 0.01).

**TABLE 4:** The effects of career optimism on organizational commitment and job—seeking behavior.

	Dependent variable						
Independent variable	Model 1 (Dependent variable: Subjective career success)	Model 2 (Dependent variable: Job-seeking behavior)	Model 3 (Dependent variable: Job-seeking behavior)	Model 4 (Dependent variable: Job-seeking behavior)	Model 5 (Dependent variable: Organizational commitment)	Model 6 (Dependent variable: Organizational commitment)	Model 7 (Dependent variable: Organizational commitment)
Constant	1.85	1.43	0.38	2.76	1.82	1.07	2.63
Career optimism	0.37^**^	0.45^**^	0.23^**^	0.21^**^	0.48^**^	0.33^*^	0.33^**^
Subjective career success			0.58^**^	0.55^**^		0.42^**^	0.36^**^
Psychological safety				0.07			0.25^**^
Subjective career success × psychological Safety				−0.10^**^			0.03
*R* ^2^	0.54	0.45	0.58	0.63	0.41	0.47	0.54
△*R*^2^	0.14	0.15	0.13	0.14	0.16	0.06	0.07
F	38.73^**^	28.73^**^	44.69^**^	41.10^**^	24.58^**^	28.94^**^	29.36^**^

*p < 0.05,

**p < 0.01.

**TABLE 5:** Results of mediation effect testing.

Mediation path	Effect	Effect value	SE	95% CI
Career optimism → subjective career Success → job—seeking behavior	Total effect	0.45	0.04	[0.36−0.53]
	Direct effect	0.23	0.04	[0.15−0.32]
	Indirect effect	0.22	0.04	[0.15−0.29]
Career optimism → subjective career success → organizational commitment	Total effect	0.48	0.05	[0.39−0.58]
	Direct effect	0.33	0.05	[0.01−0.23]
	Indirect effect	0.15	0.04	[0.15−0.23]

According to the moderated mediation procedure proposed by Edwards and Lambert (Edwards and Lambert, 2007), as indicated in Table 6, when psychological safety was set at mean ± 1 standard deviation, the difference in the indirect effect of career optimism on job-search behavior reached 0.07 (*p* < 0.01), which was significant. The test for moderated mediation showed that the index for the moderated mediation of career optimism on job-search behavior was −0.04, with a 95% confidence interval of [−0.07, −0.01], which did not contain 0. This verified Hypothesis 10.

**TABLE 6:** Results of moderated mediation effect testing.

Mediation path	Moderator	Effect size	SE	95% CI
Career optimism → subjective career success → job—seeking behavior	Low psychological safety	0.24	0.04	[0.16–0.32]
	High psychological safety	0.17	0.05	[0.07–0.26]
	Difference	0.07	−0.01	[0.06–0.09]

### 4.4 Curvilinear relationship analysis

To directly test the “double-edged sword” hypothesis of career optimism, we incorporated a quadratic term of career optimism (CO^2^) into the regression models to explore potential curvilinear relationships. The results indicated that the quadratic term of career optimism did not exert a statistically significant effect on either job-seeking behavior (β = −0.03, *p* = 0.45) or organizational commitment (β = 0.02, *p* = 0.62). This suggests that, within the range of the current data, the relationships between career optimism and the outcome variables are primarily linear, with no reversal of its positive effects observed even at higher levels. This finding supports the notion that the “double-edged sword” effect of career optimism operates more through distinct mediating pathways acting concurrently, rather than through a nonlinear direct effect.

### 4.5 Gender subgroup analysis

Given the theoretical relevance of gender in career development and stress responses, we conducted a gender subgroup analysis. The results revealed that the effect of career optimism on job-seeking behavior was significant in both males (β = 0.48, *p* < 0.01) and females (β = 0.42, *p* < 0.01), but the between-group difference was not statistically significant (Δβ = 0.06, *p* = 0.18). Similarly, the indirect effect of career optimism on organizational commitment via subjective career success demonstrated comparable patterns across both subgroups (males: ab = 0.16; females: ab = 0.14). These findings indicate that the main effects observed in this study are consistent across gender groups, providing preliminary evidence for the generalizability of the theoretical framework.

### 4.6 Sensitivity analysis

To examine the robustness of the findings, we performed the following sensitivity analyses: (1) re-estimating the models using robust standard errors; (2) re-analyzing the data after excluding extreme outliers (beyond ± 3 standard deviations); and (3) adjusting the combination of control variables. All sensitivity analyses demonstrated that the magnitude and direction of the main effects were consistent with the original analysis, and the significance patterns of the mediating and moderating effects remained unchanged. Specifically, the indirect effect of career optimism on job-seeking behavior via subjective career success remained within the range of 0.20–0.24 across all sensitivity checks, supporting the robustness of the study’s conclusions.

## 5 Discussion and implications

### 5.1 Discussion

This study explored the impact of career optimism on job-search behavior and organizational commitment, as well as the underlying process mechanisms. The findings showed that career optimism indirectly exerts a positive effect on job-search behavior and organizational commitment through subjective career success, and psychological safety can weaken the positive impact of subjective career success on job-search behavior. This study expands previous research on the relationship between career optimism and behavioral outcomes.

Eva et al. (2020) theoretically proposed that career optimism not only promotes job satisfaction and organizational commitment, but also leads individuals to generate thoughts of leaving the organization, prompting them to continuously seek new jobs; seeking promotion or new career opportunities without legitimate reasons reduces their focus on current work and lowers organizational commitment. Although scholars have attempted to explore the double-edged sword effect of career optimism from the perspective of career choice, Rottinghaus et al. found that career optimism helps individuals clarify career goals, make career choices, and enhance organizational commitment (Rottinghaus et al., 2012, 2005). In contrast, Duffy and Raque-Bogdan (2010) found that career optimism can lead to unclear career goals, thereby making it difficult to make career development decisions and increasing turnover intention and job-search behavior. The most direct consequence of career optimism is subjectively perceived career success, but the role of such subjectively perceived success is not always positive for organizations (Zacher, 2014). This study focused on career optimism, explored the mediating role of subjective career success, and clarified the moderating role of psychological safety in this process.

### 5.2 Theoretical implications

First, this study extends the conceptual discussion of the “double-edged sword” effect of career optimism proposed by Eva et al. (2020), providing empirical evidence on the key mediating mechanisms driving the coexistence of these two opposing effects. We found that subjective career success is not merely an outcome of career optimism but, more importantly, serves as a “behavioral divergence hub.” This cognitive appraisal channels positive self-perceptions simultaneously toward external opportunity exploration (job-seeking behavior) and internal loyalty reinforcement (organizational commitment). This reveals a pattern of “exploration within commitment,” in which employees are not dissatisfied with their current organization but are proactively managing their career capital—a dynamic not fully captured by traditional turnover models.

Second, the Chinese cultural context of this study may profoundly shape the variable relationships. Collectivist tendencies may strengthen organizational commitment, as employees value belonging to the organization; however, high power distance may heighten sensitivity to psychological safety, suppressing self-expression and amplifying job-seeking motivation. Guanxi networks may facilitate job-seeking behavior, as employees rely on social capital to access opportunities, while face concerns may drive individuals to seek alternative employment to preserve self-esteem in low psychological safety environments. These factors suggest caution when generalizing findings to Western individualistic cultures: the moderating effect of psychological safety on organizational commitment may be stronger (due to greater emphasis on personal expression), while job-seeking behavior may be more strongly driven by individual career goals rather than interpersonal risks. Future cross-cultural comparisons could test these hypotheses.

Compared with existing meta-analytic evidence, the effect sizes observed in this study fall within the moderate-to-strong range. For example, Eva et al.’s (2020) review of career optimism reported average correlations of *r* = 0.32–0.38 with job-seeking behavior and *r* = 0.34–0.42 with organizational commitment. In the current study, the standardized effect sizes of career optimism on job-seeking behavior (β = 0.45) and organizational commitment (β = 0.48) slightly exceed these ranges, which may reflect the heightened role of career optimism in the Chinese sample. The mediating effects of subjective career success (indirect effects of 0.22 on job-seeking behavior and 0.15 on organizational commitment) are also consistent with the meta-analytic findings of Ng et al. (2005), who reported average effect sizes of δ = 0.18–0.25 for the relationship between subjective career success and career-related behaviors. These comparisons indicate that the core relationships examined in this study are consistent in magnitude with the literature, yet slightly enhanced, suggesting that cultural or sample-specific characteristics may amplify the influence of career optimism.

Thirdly, this study expands the application of SCCT by demonstrating the dual-path mechanism through which career optimism influences external job search and internal commitment via subjective career success. The traditional Social Cognitive Career Theory framework emphasizes the direct effects of self-efficacy and outcome expectations on career behavior (Lent and Brown, 2006), yet it does not fully articulate the multiple pathways through which positive cognitions translate into actions. It is worth noting that tests for curvilinear relationships did not reveal significant nonlinear effects of career optimism, indicating that its “double-edged sword” nature manifests primarily through the simultaneous activation of both internal and external behavioral pathways, rather than through reversed effects at extreme levels.

Within the organization, career optimism leads employees to perceive greater recognition of their capabilities and value from the organization, thereby experiencing subjective career success, which in turn strengthens their organizational commitment as a form of reciprocation (Zacher, 2014).

Externally, career-optimistic employees exhibit a stronger desire to adapt to market changes through self-directed career development, gain external validation, and strive for advancement to higher platforms to achieve greater professional accomplishments (Guan et al., 2015).

By validating the core mediating role of subjective career success, this study constructs a relatively complete “cognition-appraisal-behavior” pathway. It reveals the dual-path mechanism through which career optimism, by shaping individuals’ positive perceptions of career achievements (such as competence growth and value realization), drives both external opportunity exploration (job-seeking behavior) and the maintenance of internal loyalty (organizational commitment). This finding not only aligns with the core proposition of SCCT that “cognitive beliefs influence behavior through goal appraisal” (Lent, 2013) but also reframes the theory’s static outcome variables into a dynamic transformation hub, bridging the theoretical gap between macro career attitudes and micro behavioral decisions.

Furthermore, the gender subgroup analysis indicated that the influence pattern of career optimism is similar across genders, supporting the theory’s generalizability. However, limited by sample size, the statistical power to detect between-group differences might be insufficient. Future research could further explore the potential moderating role of gender in career development.

Finally, the findings of this study significantly extend the contextual boundaries of Social Cognitive Career Theory. Although SCCT emphasizes cognitive-person factors, its insufficient consideration of organizational contextual factors has been a recognized limitation. We empirically demonstrate that psychological safety—an organizational-level climate factor—can fundamentally reshape the strength of the connection between SCCT’s core cognitive appraisal (subjective career success) and behavioral outcomes. Specifically, our research identifies psychological safety as a key boundary condition that determines whether positive self-evaluations translate into retention motivation or turnover tendency. This constitutes an important contextual extension of SCCT, enhancing its capacity to explain complex behaviors in the modern boundaryless career context.

This study extends the theoretical boundaries by demonstrating how contextual factors can reshape the strength of the relationship between cognitive appraisal and behavior, thereby addressing the theory’s longstanding neglect of situational complexity. Simultaneously, our research reframes the explanation for the “success-turnover” paradox: it offers a novel interpretation for the phenomenon of higher turnover rates among high-performing employees—specifically, in low psychological safety environments, subjective career success enhances employees’ perception of their market competitiveness, motivating them to seek safer external opportunities.

When employees perceive a high level of psychological safety, they feel empowered to express their opinions within the organization without fear of exclusion or punishment. Increased social interaction with the organization enhances their sense of organizational identification, which in turn improves performance and commitment while reducing turnover intention (Singh et al., 2013). However, this study found that psychological safety only moderates the relationship between subjective career success and job-seeking behavior, but not its relationship with organizational commitment (Hypotheses 9 and 11 were not supported).

This asymmetry may stem from the fundamental differences between job-seeking behavior and organizational commitment. Job-seeking behavior is an externally oriented, risk-sensitive action. When psychological safety is low, employees are more likely to convert subjective career success perceptions into an “escape advantage,” proactively seeking external opportunities to avoid interpersonal risks. In contrast, organizational commitment, as an internal attitude, develops through long-term organizational support, psychological contracts, and social exchange, and thus may be less sensitive to immediate fluctuations in psychological safety. Furthermore, organizational commitment might be more strongly influenced by other stable factors—such as organizational justice or leadership style—thereby weakening the moderating role of psychological safety. This finding suggests that future research should explore other boundary conditions—such as organizational identification or job embeddedness—to better explain the mechanisms underlying organizational commitment.

### 5.3 Practical implications

This study suggests that in the context of an uncertain employment environment and career development, it is particularly necessary to enhance individuals’ psychological safety while improving their career optimism.

The identified effect sizes demonstrate managerial relevance. The indirect effect of career optimism on job-seeking behavior through subjective career success (ab = 0.22) represents a medium effect size. This suggests that targeted interventions can meaningfully influence employee behavior. While the effect on organizational commitment (ab = 0.15) is smaller, it remains practically important for retaining high-value talent.

Based on the “double-edged sword” effect of career optimism and the key moderating role of psychological safety, we propose the following specific and operational suggestions for organizational managers:

Build psychological safety systematically. Train managers in providing constructive feedback, conducting blameless error reviews, and actively soliciting dissenting opinions; Implement regular anonymous pulse surveys with mandatory action plans; Establish formal blame-free debriefing mechanisms to facilitate collective learning from setbacks and failures.

Guide career optimism proactivel. Provide structured mentoring supported by evidence-based career development tools; Deliver realistic optimism that integrates market awareness, self-assessment, and achievable goal-setting.

Retain high-success employees strategically. Conduct retention-focused dialogues: “What do you need to achieve your goals here?” Grant non-monetary incentives like influence (internal expert roles) and autonomy.

In high-turnover environments, prioritize building psychological safety and create fast-track internal mobility pathways. In low-turnover settings, focus on breaking silence and designing new growth challenges to prevent complacency. Balance is essential: maintain performance standards while cultivating safety, and align career optimism with tangible internal opportunities. In practice, it is necessary to focus on comprehensively utilizing diversified methods and channels such as formal training, informal training, organizational learning, and employee self-development, and through systematic capability improvement initiatives, reserve high-quality human capital for organizations to respond to future technological changes and environmental fluctuations.

### 5.4 Limitations and future directions

This study still has certain limitations, which need to be improved in subsequent research. First, although the survey method adopted in this study did not detect common method bias, to further exclude or reduce the potential impact of this bias on the results, future studies can follow the suggestions of Podsakoff et al. (2012) and introduce objective data or multi-source data for analysis.

The study’s sample is relatively young (mean age = 30.78 years), with the majority in early career stages (58.92% having organizational tenure of 5 years or less). This demographic is typically more open to career exploration and exhibits higher external market mobility. Consequently, the “double-edged sword” effect identified in our model—particularly the strong link between career optimism and job-seeking behavior—may be especially pronounced in this group. For mid- or late-career employees, who have accumulated more organization-specific capital and often bear greater family responsibilities, career optimism might be more likely to channel into internal career development or job crafting rather than external mobility. Therefore, career stage constitutes a crucial boundary condition. Future research should formally incorporate it as a moderating variable to test and extend the boundary conditions of our theoretical model.

Furthermore, although we employed a two-wave design, it must be explicitly noted that the moderating variable (psychological safety) and the outcome variables (job-seeking behavior, organizational commitment) were measured concurrently at Time 2. This means the inferences regarding the moderating effects (H8, H9) are inherently cross-sectional, limiting our ability to draw definitive causal conclusions. While it is theoretically sound to position psychological safety as an antecedent, reverse causality (e.g., active job-seeking potentially influencing perceptions of team psychological safety) cannot be entirely ruled out logically. Future research utilizing three-wave longitudinal or experimental designs would better clarify the causal direction.

Second, while we attempted to explore more complex interaction mechanisms and subgroup differences, the sample size limited the statistical power for certain analyses (e.g., three-way interactions, some subgroup comparisons). Future studies with larger samples or experimental designs are needed to test these intricate moderating mechanisms and group differences. Additionally, this study could not directly address the issue of “concurrent behaviors”—specifically, whether the same employees simultaneously enhance organizational commitment while increasing job-seeking behavior, or whether different employees follow distinct paths. While the longitudinal design captures variable changes over time, it cannot distinguish within-person dynamics. For instance, some employees might adopt an “exploration within commitment” strategy, maintaining high commitment while job-seeking, whereas others might shift their focus across different stages. Future research could employ Experience Sampling Methodology (ESM) to track daily fluctuations or use Latent Profile Analysis (LPA) to identify employee subgroups, thereby revealing the heterogeneous behavioral patterns associated with career optimism.

Finally, the contextual breadth and variable depth of this study present avenues for future expansion. Firstly, being based on a Chinese sample, the unique cultural context (e.g., collectivist tendencies) may have shaped the mechanisms through which psychological safety and career optimism operate, suggesting that the generalizability of our findings to other cultural settings requires verification through subsequent cross-cultural comparative studies. Secondly, using job-seeking behavior rather than actual turnover as an outcome variable, while effectively capturing behavioral precursors to turnover intention, does not fully depict the complete process from cognition to final action. This process might be moderated by unmeasured factors like the external job market. Future research could adopt longer-term designs incorporating actual turnover data and macroeconomic indicators (e.g., unemployment rates) to reveal the complete mechanism from intention to action and its boundary conditions. Thirdly, the research model did not include several classic variables (e.g., pay satisfaction, promotion opportunities, person-organization fit). Their absence might affect the precise estimation of the mediating effect of subjective career success and limits the construction of a more comprehensive explanatory framework for employee retention/turnover decisions. Future studies should consider integrating these key variables to build a more holistic and powerful model of employee career dynamics.

## 6 Conclusion

The “double-edged sword” effect embodies the principle of dialectical thinking. This study focuses on career optimism, explores the “double-edged sword” effect of career optimism on job-search behavior and organizational commitment, as well as the generating mechanism of this relationship, and provides an explanatory perspective for previous research findings on contradictions in the behavioral outcomes of career optimism. Based on Social Cognitive Career Theory (SCCT), career optimism exerts an indirect positive impact on job-search behavior and organizational commitment through subjective career success, and psychological safety can weaken the positive impact of subjective career success on job-search behavior.

## Data Availability

The original contributions presented in this study are included in this article/supplementary material, further inquiries can be directed to the corresponding author.

## References

[B1] Abele A. E. Spurk D. (2009). The longitudinal impact of self-efficacy and career goals on objective and subjective career success. *J. Vocat. Behav.* 74 53–62. 10.1016/j.jvb.2008.10.005

[B2] Blau P. M. (1994). *Structural contexts of opportunities*. University of Chicago Press.

[B3] Boswell S. G. Cole B. J. Sundman E. A. Karas V. Fortier L. A. (2012). Platelet-rich plasma: A milieu of bioactive factors. *Arthroscopy* 28 429–439. 10.1016/j.arthro.2011.10.018 22284405

[B4] Boswell W. R. Roehling M. V. LePine M. A. Moynihan L. M. (2003). Individual job-choice decisions and the impact of job attributes and recruitment practices: A longitudinal field study. *Hum. Resour. Manag.* 42 23–37. 10.1002/hrm.10062

[B5] Bravo J. Seibert S. E. Kraimer M. L. Wayne S. J. Liden R. C. (2017). Measuring career orientations in the era of the boundaryless career. *J. Career Assess.* 25 502–525. 10.1177/1069072715616107

[B6] Bretz R. D. Jr. Boudreau J. W. Judge T. A. (1994). Job search behavior of employed managers. *Pers. Psychol.* 47 275–301. 10.1111/j.1744-6570.1994.tb01725.x

[B7] Carver C. S. Scheier M. F. Segerstrom S. C. (2010). Optimism. *Clin. Psychol. Rev.* 30 879–889. 10.1016/j.cpr.2010.01.006 20170998 PMC4161121

[B8] Chemers M. M. Watson C. B. May S. T. (2000). Dispositional affect and leadership effectiveness: A comparison of self-esteem, optimism, and efficacy. *Pers. Soc. Psychol. Bull.* 26 267–277. 10.1177/0146167200265001

[B9] Dearing E. Hamilton L. C. (2006). Contemporary advances and classic advice for analyzing mediating and moderating variables. *Monogr. Soc. Res. Child Dev.* 71 88–104. 10.1111/j.1540-5834.2006.00406.x

[B10] Dillard A. J. Midboe A. M. Klein W. M. (2009). The dark side of optimism: Unrealistic optimism about problems with alcohol predicts subsequent negative event experiences. *Pers. Soc. Psychol. Bull.* 35 1540–1550. 10.1177/0146167209343124 19721102

[B11] Duffy R. D. (2010). Spirituality, religion, and work values. *J. Psychol. Theol.* 38 52–61. 10.1177/009164711003800105

[B12] Duffy R. D. Raque-Bogdan T. L. (2010). The motivation to serve others: Exploring relations to career development. *J. Career Assess.* 18 250–265. 10.1177/1069072710364791

[B13] Edmondson A. (1999). Psychological safety and learning behavior in work teams. *Adm. Sci. Q.* 44 350–383. 10.2307/2666999

[B14] Edwards J. R. Lambert L. S. (2007). Methods for integrating moderation and mediation: A general analytical framework using moderated path analysis. *Psychol. Methods* 12 1–22. 10.1037/1082-989X.12.1.1 17402809

[B15] El-Anzi F. O. (2005). Academic achievement and its relationship with anxiety, self-esteem, optimism, and pessimism in Kuwaiti students. *Soc. Behav. Pers. Int. J.* 33 95–104. 10.2224/sbp.2005.33.1.95

[B16] Eva N. Newman A. Jiang Z. Brouwer M. (2020). Career optimism: A systematic review and agenda for future research. *J. Vocat. Behav.* 116:103287.

[B17] Frazier M. L. Fainshmidt S. Klinger R. L. Pezeshkan A. Vracheva V. (2017). Psychological safety: A meta-analytic review and extension. *Pers. Psychol.* 70 113–165. 10.1111/peps.12183

[B18] Gillham J. (2000). *The science of optimism and hope: Research essays in honor of martin*, ed. Seligman E. P. (Conshohocken, PA: Templeton Foundation Press).

[B19] Guan Y. Zhou W. Ye L. Jiang P. Zhou Y. (2015). Perceived organizational career management and career adaptability as predictors of success and turnover intention among Chinese employees. *J. Vocat. Behav.* 88 230–237. 10.1016/j.jvb.2015.04.002

[B20] Gunkel M. Schlaegel C. Langella I. M. Peluchette J. V. (2010). Personality and career decisiveness: An international empirical comparison of business students’ career planning. *Pers. Rev.* 39 503–524. 10.1108/00483481011045443

[B21] Hall D. T. (2002). *Careers in and out of organizations.* Newcastle upon Tyne: Sage.

[B22] Haratsis J. M. Hood M. Creed P. A. (2015). Career goals in young adults: Personal resources, goal appraisals, attitudes, and goal management strategies. *J. Career Dev.* 42 431–445. 10.1177/0894845315572019

[B23] Heslin P. A. (2005). Conceptualizing and evaluating career success. *J. Organ. Behav.* 26 113–136. 10.1002/job.270

[B24] Hobfoll S. E. (1989). Conservation of resources. A new attempt at conceptualizing stress. *Am. Psychol.* 44 513–524. 10.1037//0003-066x.44.3.513 2648906

[B25] Kahn W. A. (1990). Psychological conditions of personal engagement and disengagement at work. *Acad. Manage. J.* 33 692–724. 10.2307/256287

[B26] Kanfer R. Wanberg C. R. Kantrowitz T. M. (2001). Job search and employment: A personality-motivational analysis and meta-analytic review. *J. Appl. Psychol.* 86 837–855. 10.1037/0021-9010.86.5.837 11596801

[B27] Karrasch A. I. (2017). “Antecedents and consequences of organizational commitment,” in *Organizational commitment in the military*, ed. Gade P. A. (Hove: Psychology Press), 225–236.

[B28] Kim B. Rhee E. Ha G. Yang J. Lee S. M. (2016). Tolerance of uncertainty: Links to happenstance, career decision self-efficacy, and career satisfaction. *Career Dev. Q.* 64 140–152. 10.1002/cdq.12047

[B29] Lent R. W. (2013). Career-life preparedness: Revisiting career planning and adjustment in the new workplace. *Career Dev. Q.* 61 2–14. 10.1002/j.2161-0045.2013.00031.x

[B30] Lent R. W. Brown S. D. (2006). Integrating person and situation perspectives on work satisfaction: A social-cognitive view. *J. Vocat. Behav.* 69 236–247. 10.1016/j.jvb.2006.02.006

[B31] Lent R. W. Brown S. D. Hackett G. (1994). Toward a unifying social cognitive theory of career and academic interest, choice, and performance. *J. Vocat. Behav.* 45 79–122. 10.1006/jvbe.1994.1027

[B32] Magalhães B. C. (2015). Career optimism and organizational commitment: A study with Portuguese workers. *J. Spat. Organ. Dyn.* 3 243–254.

[B33] Meyer J. P. Becker T. E. Vandenberghe C. (2004). Employee commitment and motivation: A conceptual analysis and integrative model. *J. Appl. Psychol.* 89:991. 15584837 10.1037/0021-9010.89.6.991

[B34] Meyer J. P. Maltin E. R. (2010). Employee commitment and well-being: A critical review, theoretical framework and research agenda. *J. Vocat. Behav.* 77 323–337. 10.1016/j.jvb.2010.04.007

[B35] Ming C. Xiaoying G. Huizhen Z. Bin R. (2015). “A review on psychological safety: Concepts, measurements, antecedents and consequences variables,” in *Proceedings of the 2015 international conference on social science and technology education*, Paris.

[B36] Mitterer D. M. Mitterer H. E. (2023). The mediating effect of trust on psychological safety and job satisfaction. *J. Behav. Appl. Manag.* 23 29–41. 10.21818/001c.73642

[B37] Mowday R. T. Porter L. W. Steers R. M. (2013). *Employee—organization linkages: The psychology of commitment, absenteeism, and turnover.* Cambridge, MA: Academic press.

[B38] Newman A. Donohue R. Eva N. (2017). Psychological safety: A systematic review of the literature. *Hum. Resour. Manage. Rev.* 27 521–535. 10.1016/j.hrmr.2017.01.001

[B39] Ng T. W. Eby L. T. Sorensen K. L. Feldman D. C. (2005). Predictors of objective and subjective career success: A meta-analysis. *Pers. Psychol.* 58 367–408. 10.1111/j.1744-6570.2005.00515.x

[B40] Podsakoff P. M. MacKenzie S. B. Podsakoff N. P. (2012). Sources of method bias in social science research and recommendations on how to control it. *Annu. Rev. Psychol.* 63 539–569. 10.1146/annurev-psych-120710-100452 21838546

[B41] Podsakoff P. M. MacKenzie S. B. Lee J. Y. Podsakoff N. P. (2003). Common method biases in behavioral research: A critical review of the literature and recommended remedies. *J. Appl. Psychol.* 88 879–903. 10.1037/0021-9010.88.5.879 14516251

[B42] Rottinghaus P. J. Buelow K. L. Matyja A. Schneider M. R. (2012). The career futures inventory-revised: Measuring dimensions of career adaptability. *J. Career Assess.* 20 123–139. 10.1177/1069072711420849

[B43] Rottinghaus P. J. Day S. X. Borgen F. H. (2005). The career futures inventory: A measure of career-related adaptability and optimism. *J. Career Assess.* 13 3–24. 10.1177/1069072704270271

[B44] Seibert S. E. Kraimer M. L. Heslin P. A. (2016). Developing career resilience and adaptability. *Organ. Dyn.* 45 245–257. 10.1016/j.orgdyn.2016.07.009

[B45] Shamir-Balderman O. Shmuel H. Litaim M. (2024). The relationship among optimism, self-efficacy, occupational compromise, and happiness among young people in the post-covid-19 period. *J. Manag. Organ.* 30 2453–2473. 10.1017/jmo.2024.47

[B46] Shi Y. Cao M. (2022). High commitment work system and employee proactive behavior: The mediating roles of self-efficiency and career development prospect. *Front. Psychol.* 13:802546. 10.3389/fpsyg.2022.802546 35496228 PMC9038583

[B47] Shockley K. M. Ureksoy H. Rodopman O. B. Poteat L. F. Dullaghan T. R. (2016). Development of a new scale to measure subjective career success: A mixed-methods study. *J. Organ. Behav.* 37 128–153. 10.1002/job.2046

[B48] Simó Guzmán P. Enache C. M. Sallán Leyes J. M. Fernández Alarcón V. (2010). Analysis of the relation between subjective career success, organizational commitment and the intention to leave the organization. *Transylv. Rev. Adm. Sci.* 29E 144–158.

[B49] Singh B. Winkel D. E. Selvarajan T. T. (2013). Managing diversity at work: Does psychological safety hold the key to racial differences in employee performance? *J. Occup. Organ. Psychol.* 86 242–263. 10.1111/joop.12015

[B50] Tang Y. Shao Y. F. Chen Y. J. (2019). Assessing the mediation mechanism of job satisfaction and organizational commitment on innovative behavior: The perspective of psychological capital. *Front. Psychol.* 10:2699. 10.3389/fpsyg.2019.02699 31920781 PMC6928100

[B51] Tangirala S. Kamdar D. Venkataramani V. Parke M. R. (2013). Doing right versus getting ahead: The effects of duty and achievement orientations on employees’ voice. *J. Appl. Psychol.* 98 1040–1050. 10.1037/a0033855 23915430

[B52] Tolentino L. R. Garcia P. R. J. M. Restubog S. L. D. Bordia P. Tang R. L. (2013). Validation of the career adapt-abilities scale and an examination of a model of career adaptation in the Philippine context. *J. Vocat. Behav.* 83 410–418. 10.1016/j.jvb.2013.06.013

[B53] Wolff H. G. Moser K. (2009). Effects of networking on career success: A longitudinal study. *J. Appl. Psychol.* 94 196–206. 10.1037/a0013350 19186904

[B54] Zacher H. (2014). Career adaptability predicts subjective career success above and beyond personality traits and core self-evaluations. *J. Vocat. Behav.* 84 21–30. 10.1016/j.jvb.2013.10.002

[B55] Zikic J. Klehe U. (2006). Job loss as a blessing in disguise: The role of career exploration and career planning in predicting reemployment quality. *J. Vocat. Behav.* 69 391–409. 10.1016/j.jvb.2006.05.007

